# FMRI Reveals a Dissociation between Grasping and Perceiving the Size of Real 3D Objects

**DOI:** 10.1371/journal.pone.0000424

**Published:** 2007-05-09

**Authors:** Cristiana Cavina-Pratesi, Melvyn A. Goodale, Jody C. Culham

**Affiliations:** Department of Psychology, University or Western Ontario, London, Ontario, Canada; University of Minnesota, United States of America

## Abstract

**Background:**

Almost 15 years after its formulation, evidence for the neuro-functional dissociation between a dorsal *action* stream and a ventral *perception* stream in the human cerebral cortex is still based largely on neuropsychological case studies. To date, there is no unequivocal evidence for separate visual computations of object features for performance of goal-directed actions versus perceptual tasks in the neurologically intact human brain. We used functional magnetic resonance imaging to test explicitly whether or not brain areas mediating size computation for grasping are distinct from those mediating size computation for perception.

**Methodology/Principal Findings:**

Subjects were presented with the same real graspable 3D objects and were required to perform a number of different tasks: grasping, reaching, size discrimination, pattern discrimination or passive viewing. As in prior studies, the anterior intraparietal area (AIP) in the dorsal stream was more active during grasping, when object size was relevant for planning the grasp, than during reaching, when object properties were irrelevant for movement planning (grasping>reaching). Activity in AIP showed no modulation, however, when size was computed in the context of a purely perceptual task (size = pattern discrimination). Conversely, the lateral occipital (LO) cortex in the ventral stream was modulated when size was computed for perception (size>pattern discrimination) but not for action (grasping = reaching).

**Conclusions/Significance:**

While areas in both the dorsal and ventral streams responded to the simple presentation of 3D objects (passive viewing), these areas were differentially activated depending on whether the task was grasping or perceptual discrimination, respectively. The demonstration of dual coding of an object for the purposes of action on the one hand and perception on the other in the same healthy brains offers a substantial contribution to the current debate about the nature of the neural coding that takes place in the dorsal and ventral streams.

## Introduction

The visual processing of objects serves two major functions: object recognition and the control of object-directed actions. For example, we can distinguish between an orange and a small tangerine in a bowl of fruit, perhaps based on cues such as size. When grasping the orange however, the hand would open wider on approach than when grasping the tangerine. In both cases, we need to process visual features, and size is one of the critical features (along with shape, orientation, and slant). Goodale and Milner [1] proposed that the visual system does not construct a single representation of the world for these two different visual functions. They suggested instead that the perception of objects and the visual control of object-directed actions depend on separate streams in the cerebral cortex. According to their model, the ventral stream, in occipitotemporal cortex, transforms visual information into perceptual representations, enabling us, for example, to use relative size to distinguish the orange from the tangerine. In contrast, the dorsal stream, in occipitoparietal cortex, deals with the moment-to-moment information about the location and disposition of objects, enabling us, for example, to compute the real size of the orange to scale our grasp appropriately in flight. Here we used functional magnetic resonance imaging (fMRI) to test whether or not size-computation for action and size-computation for perception are separated in the brain.

The most compelling evidence for the two-visual-systems model comes from double dissociation studies in neurological patients [1]. Patients with lesions in the superior parietal lobe, including the intraparietal sulcus, are unable to use visual information to correctly pre-shape the hand in order to pick up objects, even though they can discriminate between objects quite normally [2]. Conversely, patient DF, who has selective bilateral damage in the ventrolateral occipital region, has no difficulty using visual information to pre-shape her hand appropriately during grasping, even though she is unable to discriminate visually amongst such objects [3]. In addition, behavioral experiments in neurologically-intact subjects have supported the proposed dissociation between vision-for-perception and vision-for-action [4], but have not addressed the specific neural substrates underlying this division of labor.

Neuroimaging, particularly fMRI, has enabled localization of specific subregions within the dorsal and ventral streams that likely subserve object recognition and object-directed action. One key region in the ventral stream is the lateral occipital complex (LOC), which is known to play a major role in object recognition by integrating visual features into object representations [5]. LOC is more activated by coherent object shapes than scrambled objects or textures [6]. One key region in the dorsal stream is the anterior intraparietal (AIP) area, which is believed to play a role in pre-shaping the hand during grasping. AIP is activated during object grasping vs. reaching [7]–[11]. Grasping requires both transport of the hand to the target and pre-shaping the hand and fingers to reflect the visual properties of the object, such as shape, size, and orientation, whereas reaching requires only transport of the hand.

The proposed roles of LOC and AIP have been supported by the combination of neuropsychological evidence and neuroimaging. One study [7] tested neurological patients with grasping deficits and found a common region of damage in the intraparietal cortex, including AIP, exactly where they also found grasping-selective fMRI activation in neurologically-intact subjects. A second study tested DF, the patient with impaired object recognition but intact grasping, and found that the main focus of her damage was located in the more lateral regions of LOC bilaterally [12]. Although DF showed no activation for line drawings of objects, she nevertheless showed activation in AIP during visually-guided grasping.

Many neuroimaging studies have explored possible dissociations between processing in the two visual streams, but these studies have used only two-dimensional (2D) stimuli and perceptual tasks. Such stimuli may not invoke dorsal stream processing as fully as three-dimensional (3D) stimuli, particularly when object-directed actions are required. With the 2D stimuli, if subjects had to process object identity, the ventral stream was engaged, whereas, if they had to process features such as spatial position, orientation, or point of view, the dorsal stream was engaged [13]–[16]. A recent fMRI adaptation study [17] used 2D videos of a grasping hand and reported that AIP in the dorsal stream was selective for both the grasp posture and the object to be grasped, whereas, the fusiform gyrus in the ventral stream was selective to the object but not the posture. One study in our lab [9] provided direct support for a dissociation between vision-for-perception in the ventral stream and vision-for-action in the dorsal stream. AIP was activated by grasping (vs. reaching) of real 3D objects but not by intact 2D images of objects (vs. scrambled objects); in contrast, LOC was activated by intact 2D images of objects but no more so for grasping than reaching. Although suggestive of a perception-action dissociation, the different pattern of activation for the two tasks in the two areas could have arisen from the different nature of the stimuli, namely 2D pictures for the perceptual task and real 3D objects for the action task. Indeed, physiological studies have reported a subset of neurons within AIP that respond to the visual presentation of real 3D objects in absence of an action [18]–[20]. Moreover, human parietal activation has been reported in the vicinity of AIP for viewing 2D pictures of tools [21] even when no action is involved. Further studies have found that this parietal activation is higher for tools than graspable and non-graspable objects, which do not differ [22], [23]. Perhaps AIP can be activated by any stimuli that have rich associations with hand actions, such as tools or perhaps real 3D objects, even though 2D objects, even graspable ones, are largely ineffective.

**Figure 1:**
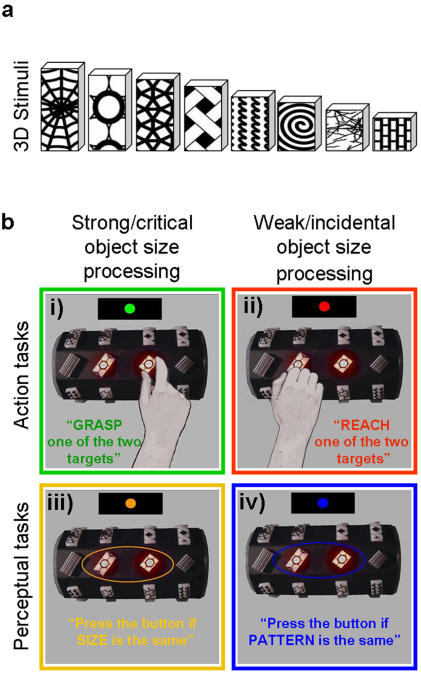
Stimuli and Experiment 1 conditions. a) Examples from the set of three dimensional objects employed for Experiments 1 and 2. In both fMRI experiments, on each trial the stimuli were the same and the tasks varied. Stimuli were 3D Plexiglas rectangles that varied in length and pattern but were of constant width and depth. b) Examples from the four conditions in Experiment 1. Subjects viewed two objects presented simultaneously and performed one of four different tasks. In the *grasping task*, subjects grasped one of the two stimuli along the long axis with the index finger and thumb. In the *reaching task*, subjects had to touch one of the two objects with their knuckles but without forming a grip. In both tasks, subjects used their right hand. On alternate trials, actions were directed to the left or the right target. For the *size discrimination task*, subjects had to decide if the two objects were the same in length by pressing a button held in their left hand. For the *pattern discrimination task* subjects had to decide if the pattern superimposed onto the two objects were the same or different by pressing the same button. In sum, we used 2×2 design contrasting action tasks with perceptual tasks, and tasks that require strong/critical size processing with tasks that required weak/incidental object size processing. In each example, the two illuminated stimuli, the grasparatus and the fixation point are shown from the subject’s point of view. Differently from the pictures, the subjects were not able to see their own hands since the experiment was performed in complete darkness (except for the illumination of the fixation point).

In Experiment 1, we examined whether or not a dissociation between dorsal and ventral stream areas could still be demonstrated when the same 3D objects were used both in a grasping task and in a perceptual judgement task (Fig. 1). We employed two perceptual discrimination tasks and two visually-guided action tasks. For one perceptual task, size discrimination, size computation was critical for the task; for the other perceptual task, pattern discrimination, size computation was negligible or incidental. For one action task, grasping, the computation of object properties, including size, was critical for the task; for the other action task, reaching, size computation was negligible or incidental. We cannot rule out the possibility that some incidental size computation was occurring in pattern discrimination or reaching (for example, reaching may be less demanding when directed to larger targets); however, in both cases, size computation would be much weaker than required in size discrimination and grasping. Unlike previous fMRI studies comparing vision for action and vision for perception, we kept the stimuli constant and varied the tasks accordingly to their ability to recruit the dorsal (grasping and reaching) or ventral (discrimination of size and pattern) streams. We hypothesized that AIP (within the dorsal stream) would be modulated by size processing but only for the action task, whereas LOC (within the ventral stream) would be modulated by size processing but only for the perceptual task.

We also performed a control experiment (Experiment 2) to examine whether or not AIP would be activated by the visual presentation of a 3D object in the absence of a task. Given the physiological data from the macaque, we hypothesized that AIP would be activated by passive viewing of 3D objects even in the absence of an action.

The critical questions addressed by these experiments are (i) whether AIP shows a response to the visual presentation of 3D objects when no action is required; and (ii) whether this response is modulated by the computation of size for perception of 3D objects. Although our prior study [9] suggested that 2D objects did not drive AIP very strongly, it is quite possible that AIP may be specialized for 3D object processing, regardless of the nature of the task. That study already demonstrated that LOC does not respond to size processing during grasping (vs. reaching). With respect to size processing during perception, there are reasons to expect that a preference for computation of global size vs. local texture might be observed at least within a subdivision of LOC, if not the entire complex. Past studies have suggested that the LOC may comprise several functional subregions, though as yet, there is little consensus about the number and nature of those subregions [5], [24]–[27]. Recent work from our group and others has led to the proposal that the lateral portion of LOC may be involved in the perceptual processing of global object properties such as shape, orientation and size [12], [28], [29] whereas the ventral occipitotemporal portion of LOC (VOT) may be more concerned with material properties such as colour, texture and pattern [12]. Based on these results, we hypothesized that the lateral subdivision of LOC (sometimes referred to simply as LO) would be more responsive during a perceptual discrimination of global size than local pattern. Although the demonstration of a greater activation for size discrimination (over pattern discrimination) within LOC, or a subdivision of it, was not a central to our main question about the responsiveness of AIP to 3D objects, it was still valuable in that it could provide evidence for a dissociation. That is, the simple demonstration that AIP did not participate in perceptual size discrimination (vs. pattern discrimination) would be much more convincing if the contrast between the two tasks was sensitive enough to produce activation for this discrimination in another visual area, particularly a ventral-stream area such as LO.

## Materials and Methods

### Subjects

Ten young (age range: 22–33) university students (six female) participated. They were all right-handed as measured by the Edinburgh Handedness Inventory [30]. All participants provided informed consent before beginning the experiment which was approved by the Health Sciences Research Ethics Board at the University of Western Ontario. All ten subjects performed repeated functional runs for experiment 1 and experiment 2 as well as one anatomical scan during the same session. One subject was not included in the analysis due to methodological problems during data acquisition and the presence of strong head motion.

### Apparatus

Perceptual discrimination and goal-directed action tasks were performed by the use of the “grasparatus” grasping apparatus [9], [31], a metal-free device which allow a computerized presentation of a wide range of real 3D stimuli. The grasparatus consists of an octagonal rotating drum with four translucent rectangular 3D shapes on each of eight faces. A super-bright red light-emitting diode (LED; 12 candelas/m^2^) was located beneath each of the four target locations facing the subject. The grasparatus could be rotated to each of the eight faces between trials using a computer-controlled pneumatic system [see 9, for details].

During the experiments, the subjects laid supine within the magnet with the torso and the head tilted at an angle (∼30 deg) that permitted direct viewing of the stimuli, without mirrors. Such direct viewing, which has only been used in two prior studies examining visually guided grasping [7], [31], avoids introducing additional transformations required by mirror-viewing [32] The grasparatus was placed approximately 10 cm above the subject’s pelvis in order to present the stimuli at a comfortable and natural grasping distance. Subjects lay in the magnet in complete darkness (to reduce activation due to motion of the hand) with the right hand placed at a starting position around the navel and the left hand placed beside the body holding a response button. They were asked to maintain fixation on the light-emitting diode (LED, masked by a 0.1° aperture) mounted on the ceiling of the bore for the duration of all experiments. The LED could be illuminated in one of four different colors (green, red, yellow and blue) to indicate the task for each trial. Because of the torso and head tilt, the natural line of the gaze toward the fixation LED was approximately 10° of visual angle above the grasparatus. This positioning was chosen in order to avoid discomfort from continual gazing downward toward the grasparatus. Thus, the stimuli were presented in the lower visual field of the subject, a common configuration in the everyday interaction with objects. In order to minimize head movement related to the action tasks, a hemi-cylindrical arm brace with Velcro straps supported and restrained the subject’s right upper arm. The arm brace allowed full motion of the wrist (in order to grasp and reach any object orientation comfortably), limited motion at the elbow (enough to move the lower arm from the resting position toward the stimuli) and no motion at the shoulder. All of the hardware (LEDs and a pneumatic solenoid) and the software (VisionShell software) were triggered by a computer (Macintosh G4) that received a signal from the MRI scanner at the start of each trial.

### Stimuli

A set of 32 3D objects were used for both the action and the perceptual tasks (Fig. 1a). Stimuli were translucent white plastic rectangles of constant width (1.6 cm) and depth (0.6 cm) but with varying length (1.9–4.0 cm, steps of 0.3 cm). We used regular geometric shapes rather than functional objects (i) for comparability with macaque neurophysiology studies [18]–[20], [33]; and (ii) to examine grasping generally rather than the left-hemisphere network specialized for functional objects such as tools [34]. Care was taken to arrange the orientation (vertical, horizontal and oblique) of all objects such that they could be comfortably grasped with a precision grip. The face of each object was covered by a pattern. Each pattern was chosen from CorelDraw (Version 11, 2002) and printed onto transparent adhesive labels (some of the patterns are shown in Fig. 1a). Each label was then mounted on the object face and cut precisely along the edges. The four objects on each face were chosen to enable paired combinations of two objects that could have the same pattern and length, the same pattern but different lengths, the same lengths but different patterns, or different patterns and lengths. Although sizes varied from face to face, the size difference was always 0.6 mm. The two patterns paired on any given face had comparable overall luminance levels (to ensure that any effects of pattern discrimination were not due to luminance confounds).

### Tasks procedures

#### Experiment 1

Two stimuli were illuminated for 500 ms simultaneously on each trial and subjects performed one of four possible tasks depending on the color of the fixation point. If the fixation point was green, subjects grasped one of the two objects; if it was red, subjects reached toward one of the two objects; if it was yellow, subjects discriminated between the sizes of the two objects; and if it was blue, subjects discriminated between the patterns of the two objects. In the grasping condition (G), subjects transported their arms to the target location and grasped along the vertical axis of the rectangular shape using a precision grip with their index and thumb. The objects were firmly mounted on the cylinder, so the subjects did not attempt to lift them. In the reaching condition (R), subjects transported their arms to the target location, but rather then forming a grip, they simply touched the objects with their knuckles. This form of reaching, instead of directing the index finger toward the center of the object, was chosen to avoid any shape processing that might be necessary to compute the centroid of the target. After each grasping or reaching action, the subjects returned the hand to the starting position and waited for the next trial to start. Since two stimuli were presented simultaneously, we asked the subjects during grasping and reaching, to act alternately toward the rightward or leftward object of the pair. In the size discrimination condition (S), the subjects were instructed to press a button if the two objects shared the same length and withhold the button press if the two objects differed in length. In the pattern discrimination condition (P), the subjects were instructed to press the button if the two objects shared the same pattern and withhold the button press if the two objects differed in pattern. To reduce cognitive demands caused by frequent task changes, we employed a slow event-related design with trials spaced every 14 s in short blocks of four trials that alternated among conditions (e.g., RRRRGGGGPPPPSSSSGGGG…). Each run was structured in a series of 8 blocks of tasks (with 2 blocks of each task) with 4 trials per block, for a total of 32 trials (8 minutes) per run. Each subject performed a minimum of 4 runs for at least 36 trials per task.

#### Experiment 2

Tasks procedures were almost identical to Experiment 1 with the difference that we illuminated only one stimulus at a time and the presentation duration was shorter (250 ms). Subjects were asked to Grasp (G) or Reach (R) if the fixation point was green or red, respectively, following the procedures illustrated in Experiment 1. As a third condition, if the fixation point was blue, subjects were asked to passively view (V) the stimulus without moving their arms or making any stimulus discrimination. The event-related timing was identical to Experiment 1, but the sequence of trials was randomly intermingled instead of organized in blocks (e.g., GRVRGVGVR…). Each condition was repeated 9 times for a total of 27 trials and an overall run time of 7 minutes. Each subject performed at least 2 runs for at least 18 trials per condition.

### Imaging parameters

All imaging was performed at the Robarts Research Institute (London, ON, Canada) using a 4-Tesla whole body MRI system (Varian, Palo Alto, CA; Siemens, Erlangen, Germany). A transmit-receive, cylindrical birdcage radiofrequency head coil was used in all experiments. Each scan session consisted of at least 6 functional runs and a high-resolution anatomical scan. BOLD-based [35] functional MRI volumes were collected using an optimized segmented T2*-weighted segmented gradient echo echoplanar imaging (19.2 cm field of view with 64×64 matrix size for an in-plane resolution of 3 mm, repetition time (TR) = 1 s with two segments/plane for a volume acquisition time of 2 s, time to echo (TE) = 15 ms, flip angle (FA) = 45 deg, navigator-corrected). Each volume comprised 14 contiguous slices of 6mm thickness, angled at approximately 30 deg from axial to sample occipital, parietal, posterior temporal and posterior/superior frontal cortices. A constrained 3D phase shimming procedure was performed to optimize the magnetic field homogeneity over the prescribed functional planes [36]. During each experimental session, a T1-weighted anatomic reference volume was acquired along the same orientation as the functional images using a 3D acquisition sequence (256×256×64 matrix size, 3.0 mm reconstructed slice thickness, TI = 600 ms, TR = 11.5 ms, TE = 5.2 ms, FA = 11 deg).

### Data Analysis

We used the Brain Voyager 2000 software package (Brain Innovation, Maastricht, The Netherlands) for data analysis. Functional data were superimposed on anatomical brain images, aligned on the anterior commissure–posterior commissure line, and transformed into Talairach space [37]. Functional data were preprocessed with temporal high-pass filtering (to remove frequencies below 3 cycles per run). Data were analyzed using a General Linear Model (GLM) with separate predictors for each trial type. The model included four predictors for Experiment 1: Grasping, Reaching, Size discrimination and Pattern discrimination and three predictors for Experiment 2: Grasping, Reaching and Passive viewing. Predictors were modeled beginning with a 2s (or 1 image volume) rectangular wave for each trial. This time window was chosen because it covered stimulus presentation and subject response (for both action and button press). The remaining 12 s were considered as the intertrial interval (ITI). Each predictor was then convolved with a standard hemodynamic response function.

Artifacts related to the motion of the arm at the start of each trial were removed. Consistent with field distortion artifacts from the moving arm [38], [39] we detected positive and/or negative spikes of approximately 1% signal change in the first volume of each trial. We attributed those artifacts to the distortion of the magnetic field due to the changing position of the arm during the action tasks [38]. The employment of an event-related design enabled us to dissociate spurious signal change from true activations. While artifacts occur without a delay, true activations occur at the standard hemodynamic lag of approximately 5 s and with the characteristic hemodynamic response profile. We eliminated the artifacts by removing the first volume of every trial by using Matlab v 6.1 (The MathWorks, Natick, MA) and by adjusting the hemodynamic response accordingly. 

We used multiple criteria to check for possible motion artifacts in every run of every subject. First, we viewed cine-loop animations of all functional runs to ensure no visible movements or artifacts. Second, we ran each run through a motion correction algorithm and evaluated the output for the signatures of corrupting artifacts (after removal of the hand motion artifacts described above), particularly abrupt changes in the motion parameters within a run. Third, given that even small motions can lead to artifactual activation if correlated with the paradigm [40], we also carefully inspected activation maps for each subject individually to ensure no artifactual activations at tissue boundaries. Substantial head motion artifacts were observed in one subject whose data were consequently excluded from the analyses. In the remaining subjects, all of whom were highly experienced, there was negligible head motion indicated by the three criteria employed. We chose to analyze the uncorrected rather than the motion corrected data. Although some have recently suggested that motion correction [41] and/or inclusion of motion parameters as covariates [42] can improve cluster size and significance for data from first-time subjects at 3 Tesla, other studies have found that motion correction can actually make the data worse [39]. While motion correction algorithms correct for artifacts due to changes in head position, they do not correct for distortions of the magnetic field caused by the moving mass of the head (or in our experiments, movements of the limb). These field distortion artifacts are more pronounced at high field strengths such as our 4 Tesla scanner and can severely mislead motion correction algorithms [43]. In cases where we investigated the effect of motion correction on our grasping data, we have observed negligible improvement [44]; hence, we chose to evaluate the uncorrected but carefully screened data from highly experienced subjects.

We performed two types of analyses on the neuroimaging data. First, because we had definite hypotheses about two specific areas, AIP and LOC, we began by identifying each of those two areas within single subjects using a region of interest (ROI) approach. Second, to examine whether or not additional areas beyond AIP and LOC would display interesting activation patterns, we performed voxelwise contrasts between conditions in our group data averaged in stereotaxic space [37].

For the ROI analysis, regions were defined in each individual by contrasting conditions in Experiment 1, using a threshold of p<0.001, uncorrected (except in two subjects, where the threshold was p<0.01). In the voxelwise analysis, statistical activation maps were set to reliable threshold levels and cluster volumes (p <.001, minimum cluster size = 108 mm^3^) using Monte Carlo simulations (performed with AlphaSim software, courtesy of Douglas Ward, Medical College of Wisconsin) to verify that our regions of interest were unlikely to have arisen due to chance, given the problem of multiple comparisons.

For each selected area (in the single subject ROI approach and in the averaged voxelwise approach) we extracted the event-related time course for each subject, each condition and each experiment separately. We extracted the average percent signal change during the peak three volumes (4–8 s after each trial began) and applied Multivariate Analysis of Variance (MANOVA) using TASK (visuomotor and perceptual) and OBJECT FEATURE (size vs. non-size) as main factors. We then performed paired sample t-tests for post-hoc comparisons between conditions and one sample t-tests for comparisons of activation with respect to the baseline (computed as the last volume of the ITI period which was set to zero).

## Results

### Behavioral accuracy

To ensure that the size and pattern discrimination tasks in Experiment 1 were of comparable difficulty, we analyzed response accuracy. Overall accuracy was high and there was no difference between tasks (size = 75% and pattern = 78%; p = 0.301). Nevertheless, there were more ‘same’ responses in the size discrimination task than in the pattern discrimination task (i.e., more button presses for the size discrimination task, p = .01).

### Region of Interest analysis across individual subjects

AIP was identified by a contrast between grasping vs. reaching in Experiment 1 [7]–[10]. LOC was identified by a contrast between size vs. pattern discrimination in Experiment 1. Typically, LOC has been defined by a comparison between intact 2D images of objects vs. their scrambled counterparts. This type of contrast was not possible with our 3D objects. Nevertheless, given the putative role of LOC in extracting object form rather than material properties [12], [45], we expected that LOC would be more active when subjects attended to the global form-specific dimension of size than to the local superimposed pattern. Once each area had been defined, we extracted activation time courses and computed the peak activation (percent BOLD signal change, %BSC) for each of the four conditions. We evaluated whether or not each area also showed a significant differential response in the independent contrast. Specifically, we evaluated whether AIP and LO would show a significant, but opposite, interaction between TASK and OBJECT FEATURE. A functional dissociation between the two areas would predict that AIP, defined by grasping versus reaching, would not show a significant activation difference between size and pattern discrimination. Conversely LO, defined by size vs. pattern discrimination, would not show a significant activation difference between grasping and reaching.

In addition, for each ROI localized by Experiment 1, we extracted the BOLD response for each of the conditions in Experiment 2 so that we could examine the response to the passive viewing condition as well.

#### Anterior Intraparietal Area

**Figure 2:**
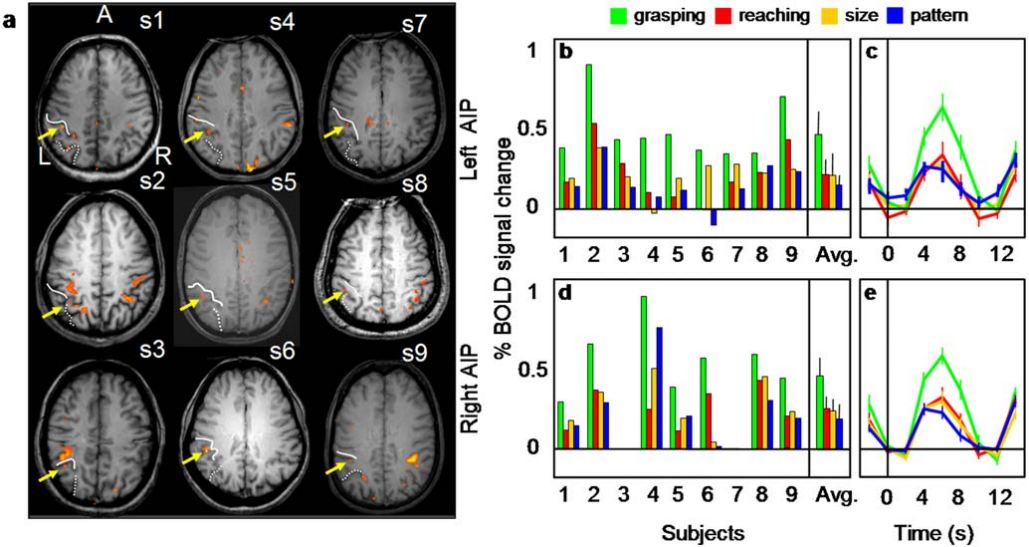
Individual activation maps and % BOLD signal change (% BSC) in the anterior intraparietal (AIP) sulcus for Experiment 1. a) The exact position of the left AIP localized by comparing grasping versus reaching is shown in the most clear transverse slice for each of the nine subjects. In each subject, AIP (highlighted by a yellow arrow) lay at the junction of the anterior end of the intraparietal sulcus (dotted line) and the inferior segment of the postcentral sulcus (PCS - plain line). Note that in five of the nine subjects AIP is also visible in the right hemisphere. b,d) Bar graphs display the magnitude of peak activation in % BSC in each experimental condition at the level of single subject and group average (the rightmost bars) for left and right AIP. For two of the nine subjects, the peak activation was not computed in the left AIP because no activity was found in that area. c,e) Line graphs indicate the event-related averaged time course in % of BSC for the four experimental conditions in the right and left AIP with time zero indicating the onset of the visual stimuli. Both magnitude of peak activation and event-related averaged time courses show that activation for grasping was higher than that for reaching and that the activation associated with size discrimination did not differ from that associated with pattern discrimination. L = left, R = right, P = posterior.

AIP was localized in each participant by selecting the voxels located in the anterior part of the intraparietal sulcus (IPS) that were significantly more active on grasping vs. reaching trials. We located a focus of activation at the junction of the anterior IPS and the postcentral sulcus (PCS) in the left hemisphere of all nine subjects and in the right hemisphere of seven subjects. In cases where the postcentral sulcus is interrupted, dividing it into an inferior and superior portion, AIP is reliably at the junction of the inferior PCS and the IPS (approximately 55% of cases, [46]); in cases where the PCS is continuous (the remaining ∼45% of cases), AIP is reliably at its junction with the IPS. There was also a consistent cluster of voxels located more posterior along the horizontal segment of IPS [47] in the left hemisphere of eight subjects and the right hemisphere of five subjects. In each subject, we concentrated our analysis on the more anterior focus (left X = −40; Y = −41; Z = 39 and right X = −35; Y = −42; Z = 44) rather than the posterior focus (left X = −28; Y = −58; Z = 41 and right X = 25; Y = −64; Z = 42) because the anterior focus was in better agreement with coordinates from previous fMRI experiments that used real 3D stimuli [7], [9], [10], [48]–[50]. The more posterior focus along the IPS might be the caudal intraparietal area, CIP, which has been shown to be activated in orientation discrimination tasks with three dimensional stimuli [51]. Left AIP activation is shown for each participant in Figure 2a together with the average time courses and peak activation from left and right AIP (Fig. 2b–e).

Having defined AIP by contrasting grasping vs. reaching, we then examined the relationship between TASK and OBJECT FEATURE in term of %BSC for Experiment 1. We found a significant interaction between TASK and OBJECT FEATURES (left AIP: F(1,8) = 26.063, p = 0.001; right AIP: F(1,6) = 11.336, p = 0.015) showing that AIP responded higher for grasping than reaching (left AIP, p = 0.0001 and right AIP, p = 0.01) but, most critically, it did not show a significantly greater response to size than pattern discrimination (left AIP p = 0.2, and right AIP p = 0.7). Additional post hoc t-tests indicated that grasping was higher with respect to all the other conditions (p< 0.01 for all comparisons) which did not differ from each other (p>0.05 for all comparisons). Activation in each of the four conditions was significantly greater than baseline activity (p<0.01 for left AIP and p< 0.05 for right AIP).

**Figure 3:**
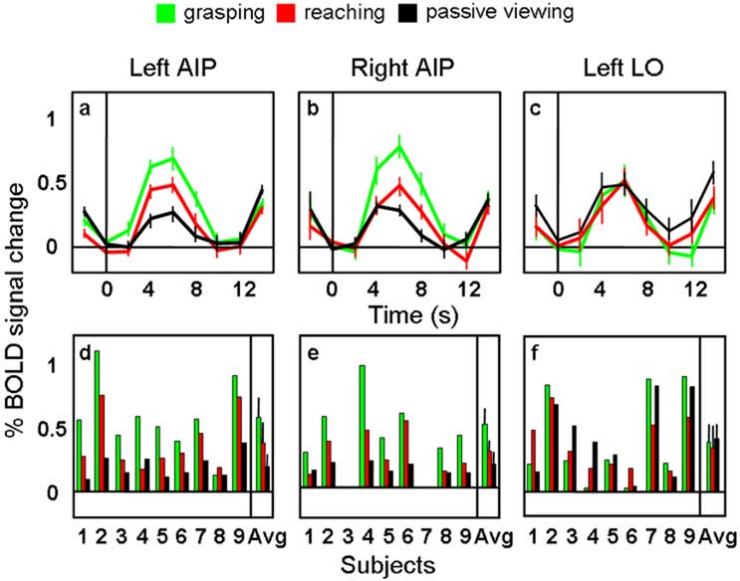
Percent of BOLD signal change (% BSC) for Experiment 2 in the left AIP (a,d), in the right AIP (b,e) and in the left LO (c,f) localized by Experiment 1. **a,b,c**) Line graphs indicate the event‐related averaged time courses in % BSC with time zero indicating visual stimuli onset. **d,e,f**) Bar graphs display the magnitude of peak activation in %BSC in each of the three experimental conditions at the level of single subject and group average (the rightmost bars). Right and left AIP (**a,b,d,e**) responded to grasping, reaching, and passive viewing, even though higher for grasping. LO (**c,f**) was equally activated by grasping, reaching, and passive viewing.

In Experiment 2, we evaluated whether or not AIP, as localized in Experiment 1, would be activated by passive viewing of 3D objects (Fig. 3a,b,d,e). Indeed, in both hemispheres, the AIP response to passively viewed objects was significantly greater than baseline (left AIP p<0.05 and right AIP p<0.001). However, activation during passive viewing was significantly lower than activation during both grasping (left and right AIP, p<.001) and reaching (left AIP, p<.01; right AIP, p<.05). Not surprisingly, the activation associated with grasping in Experiment 2 was significantly higher than for reaching in both hemispheres (p<.01).

**Figure 4:**
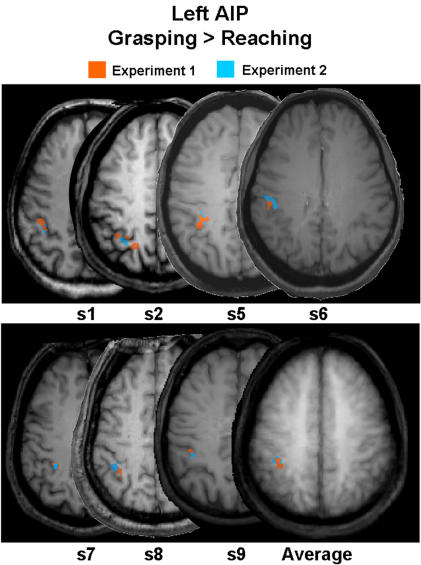
Overlaid activation maps for AIP in Experiment 1 and Experiment 2. Selected clusters of voxel activity for grasping versus reaching in the vicinity of AIP for Experiment 1 (depicted in orange) and for Experiment 2 (depicted in light blue) are overlapped on the same axial slice for each subject and for the averaged group data (rightmost slice). Data for two of the nine subjects (s3 and s4) could not be reported because the passive viewing condition was included in Experiment 1 rather than Experiment 2.

We wanted to check if the localization of AIP was reliable across experiments. Therefore we performed a subtraction between grasping and reaching for Experiment 2 and compared it to the same subtraction based on the data from Experiment 1. The subtraction of grasping versus reaching in Experiment 2 revealed activation in the majority of subjects (seven of nine) in a location that overlapped very well with AIP from the same subtraction in Experiment 1 (averaged stereotaxic coordinates for left X = −38; Y = −45; Z = 44 and for right X = 39; Y = −44; Z = 43), despite the fact that fewer runs were collected in Experiment 2. Figure 4 shows the overlap between activated voxels in AIP for the contrast of grasping versus reaching in Experiment 1 (depicted in orange) and Experiment 2 (depicted in light blue), plotted on the same sagittal slice for each subject.

We do not think that the pattern of activation we found in AIP reflects specific kinematic differences between grasping and reaching. It might be argued, for example, that grasping, in comparison to reaching, is likely to take more time because of greater coordination demands. In order to rule out this hypothesis we used an opto-electronic recording system (Optotrak; Northern Digital, Waterloo, ON) to collect kinematic data outside the magnet in conditions as similar as possible to the scanning sessions. Of the nine subjects who originally participated in the experiment, only four were available for the control experiment. Subjects were asked to lie on the floor with the head tilted and to reach (with the knuckles) or grasp (with the index and the thumb) the 3D objects mounted on the grasparatus while the positions of the index finger and thumb were recorded. As in the magnet session, subjects were asked to fixate above the 3D stimuli, which were visible for 500 ms. Subjects were not told about the purpose of the control experiment and were simply asked to perform the movements as they had in the fMRI experiment. We used movement duration (MD) as main dependent measure. Movement onset and offset were defined as the point at which velocity went above and below (respectively) 20 mm/s for more then 10 consecutive frames respectively. There was a negligible difference in MD for the grasping (603.9 ms) vs. reaching (604.8 ms) actions, a difference that was nonsignificant (χ^2^ = 1; p = .317) in a non-parametric test (Friedman test) suitable for the small sample size. These four subjects, like the other five, all showed a higher AIP response for grasping vs. reaching despite the absence of differences in movement duration.

#### Lateral Occipital Cortex

**Figure 5:**
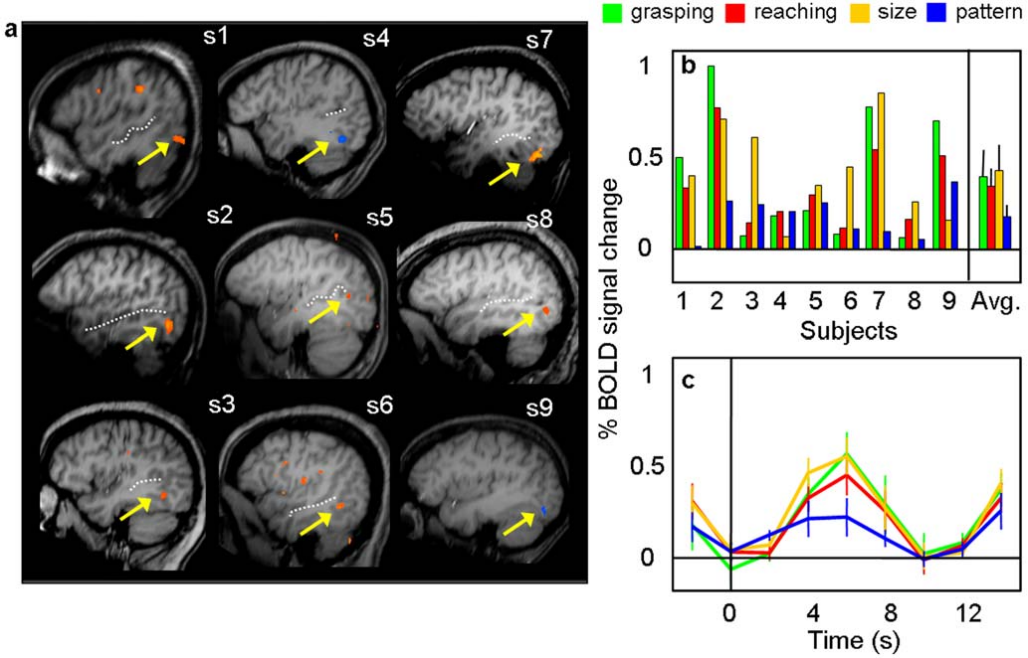
Individual activation maps and percent BOLD signal change (% BSC) in the left lateral occipitotemporal (LO) cortex for Experiment 1. **a)** The exact position of the left LO localized by comparing size discrimination versus pattern discrimination is shown in the clearest sagittal slice for each of the nine subjects. In each subject, LO was below the posterior end of the inferior temporal sulcus (ITS, dotted line). Note that in 2 of the 9 subjects, LO was activated by the reverse contrast of pattern versus size discrimination (reported in blue). **b**) Bar graphs display the magnitude of peak activation in %BSC in each experimental condition at the level of single subject and group average (the rightmost bars). **c**) Line graphs indicate the event‐related averaged time courses in % BSC with time zero indicating visual stimuli onset. Both magnitude of peak activation and event‐related averaged time courses showed that size discrimination was higher than pattern discrimination and moreover that grasping did not differ from reaching.

A comparison of size discrimination vs. pattern discrimination revealed a focus in the left lateral occipitotemporal cortex in seven of nine subjects. As shown in Figure 5a, the activation was typically below the posterior end of the inferior temporal sulcus (ITS, dotted line in the figure). The average stereotaxic coordinates (X = −46; Y = −67; Z = −8) were congruent with the suggested location of the lateral subdivision of LOC, often called LO in previous literature [e.g. 27]. It is this lateral subdivision that has been implicated in the perception of global shape properties, in contrast to the ventral occipitotemporal subdivision, which has been implicated in local object characteristics [12], [28], [29]. In two of the nine subjects, the reverse contrast, between pattern vs. size discrimination, revealed activation in the same vicinity (shown in blue for subjects 4 and 9). It is unclear why the reverse pattern was observed in these subjects. It is possible that they used a different strategy to perform the tasks, for example, relying more on local shape rather than the overall pattern during pattern discrimination. The average activation time course is shown in Figure 5b,c. Analysis of the %BSC showed a significant interaction for TASK and OBJECT FEATURE (F(1,8) = 8.026; p = 0.022) showing that LO responded more highly to size versus pattern discrimination and, most critically, did not show a significantly greater response to grasping than reaching, regardless of whether we included data from the two subjects with the reverse activation pattern (p = 0.28). The activation for size discrimination did not differ significantly from grasping (p = .79) or reaching (p = .34). Activation in each of the four conditions was significantly greater than the baseline activity (p<.01).

In Experiment 2 we evaluated whether or not LO, as localized by Experiment 1, would be activated by passive viewing of 3D objects (Fig. 3c,f). As expected, the LO response to passively viewed objects was significantly greater than baseline (p< .003) and did not differ from grasping and reaching (p>0.4).

### Voxelwise Analysis for Group Data

**Figure 6:**
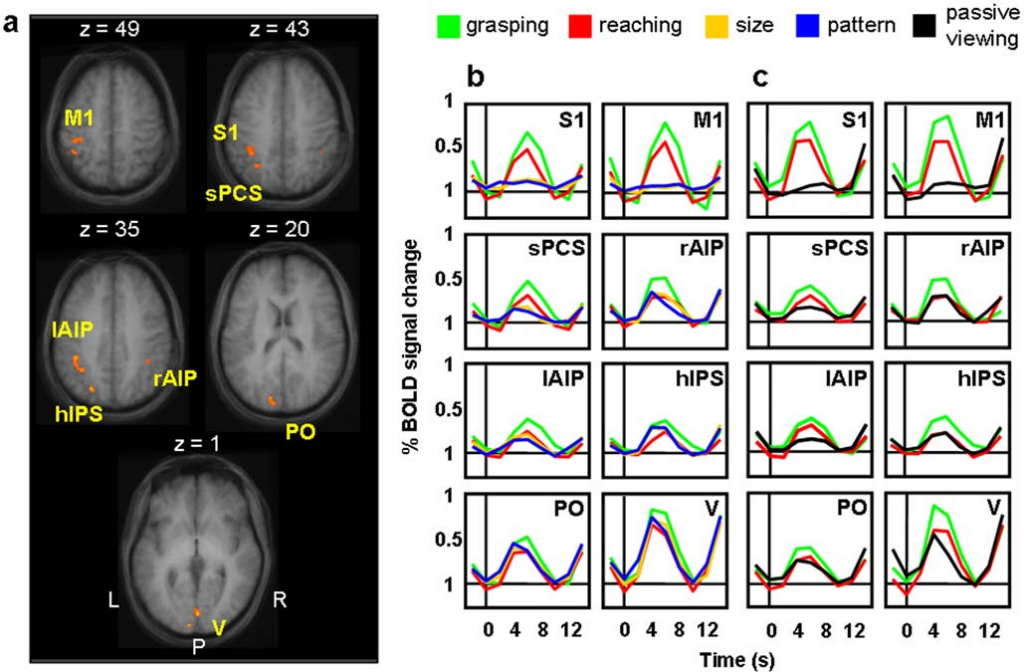
Group activation maps and percent BOLD signal change (% BSC) for grasping minus reaching in Experiment 1 and Experiment 2. **a)** Brain areas activated by comparing grasping vs. reaching in Experiment 1: left primary motor cortex (M1, within the central sulcus), left primary somatosensory cortex (S1, within the postcentral sulcus), the left superior postcentral sulcus (sPCS), the left and right anterior intraparietal sulcus (lAIP and rAIP), the left horizontal segment of the intraparietal sulcus (hIPS), the parieto‐occipital cortex (PO) and early visual cortices (V). The group activation map is based on the Talairach averaged group results shown on group‐averaged. **b**) Event‐related averaged time courses measured in each area for Experiment 1. **c**) Event‐related averaged time courses for Experiment 2 in each of the brain regions localized by Experiment 1. Brain activation is measured in % BSC and time zero indicates visual stimuli onset. For both experiments, Talairach coordinates for the activated areas and p values for the relevant statistical comparisons are shown in Table 1. L=left, R=right, P=posterior.

The voxelwise comparison of grasping vs. reaching in Experiment 1 produced activations in AIP bilaterally, as well as in several additional areas of the left hemisphere (Fig. 6a). Additional activation was observed in primary motor cortex (M1, within the central sulcus), primary somatosensory cortex (S1, within the postcentral sulcus), the superior postcentral sulcus (sPCS), the horizontal segment of the intraparietal sulcus (hIPS), parieto-occipital (PO) cortex and early visual cortices (V). Most critically, no activation was found in the vicinity of LO. As shown in Figure 6b, in each area, the % BSC is comparable to the pattern of results found in AIP using the ROI approach. That is, a significant interaction was found between TASK and OBJECT FEATURES (see table 1 for statistical values) where grasping activation was higher than reaching, but there was no significant difference between activation in size vs. pattern discrimination trials. In all areas, except M1 and S1, activations for grasping, reaching, size discrimination, and pattern discrimination were all significantly higher than baseline. In Experiment 2, all areas defined in Experiment 1 showed greater activity for grasping vs. reaching, and all areas, except M1 and S1, showed significant activity for passive viewing compared to the baseline (Fig. 6c). For both experiments, the activation in S1 and M1 for the perceptual conditions (size discrimination, pattern discrimination and passive viewing) did not differ from the baseline. Talairach coordinates for each area and p values for each statistical comparison for both experiments are shown in Table 1.

**Table 1:** Areas significantly active for the voxelwise comparison of grasping versus reaching and size versus pattern discrimination in Experiment 1.

Regions	Talairach Coordinates			Volume mm^3^	% BSC Experiment 2							% BSC Experiment 2					
					One-Sample t-test				Interaction (MANOVA) Paried-Sample t-test			One-Sample t-test			Paried-Sample t-test		
	*x*	*y*	*z*		G	R	S	T	T×Of	G>R	S>P	G	R	P	G>R	G>P	R>P
Grasp>Reach																	
Left CS	−34	−28	52	164	*	*	-	-	0.018	0.013	0.491	*	*	-	0.001	0.0001	0.0001
Left PostCS	−36	−39	45	479	*	*	-	-	0.010	0.004	0.290	*	*	-	0.003	0.0001	0.001
Left SPC	−29	−53	43	429	*	*	*	-	0.015	0.002	0.829	*	*	*	0.006	0.014	0.169
Right AIP	39	−39	38	294	*	*	*	*	0.008	0.001	0.255	*	*	*	0.001	0.0001	0.146
Left AIP	−37	−42	36	311	*	*	*	*	0.031	0.001	0.15	*	*	*	0.011	0.008	0.032
Left hIPS	−22	−68	35	169	*	*	*	*	0.022	0.027	0.864	*	*	*	0.001	0.017	0.787
Left PO	−12	−84	20	200	*	*	*	*	0.001	0.003	0.99	*	*	*	0.002	0.016	0.665
Visual Areas	0	−79	8	599	*	*	*	*	0.011	0.001	0.262	*	*	*	0.001	0.0001	0.026
Size>Pattern																	
Right SMA	5	0	48	263	*	*	*	*	0.023	0.218	0.002	*	*	-	0.314	0.128	0.693
Right IPL	46	−43	43	282	*	*	*	*	0.004	0.141	0.006	*	*	-	0.143	0.020	0.095
Left pIPS	−29	−88	6	335	*	*	*	-	0.042	0.915	0.008	*	*	*	0.498	0.499	0.927
Left LO	−49	−65	−9	185	*	*	*	*	0.001	0.677	0.015	*	*	*	0.88	0.969	0.534

For each area, Talairach coordinates, volume in mm^3^ and statistical significance for t-tests comparisons are reported. Experiment1: G = grasp; R = reach, S = size discrimination; P = pattern discrimination. Experiment2: G = grasp; R = reach; P = passive viewing; T×Of = Task×Object features. (*) = significantly different from the baseline; (-) = not significantly different from the baseline.

The negligible, nonsignificant activation for size discrimination, pattern discrimination, and passive viewing conditions in S1 was used as a reliable marker to tease apart AIP from the nearby somatosensory areas located in the vicinity of the PCS. Although S1 responded more to grasping than reaching, perhaps because of the greater tactile stimulation of the fingers during object contact [31], only AIP showed a visual response above baseline for the three non-action conditions (size discrimination, pattern discrimination and passive viewing).

It is unlikely that the bilateral activation we observed in AIP was due to button presses in the two discrimination tasks, which would be expected to activate contralateral somatosensory and motor cortex. Nevertheless, to verify that the response in AIP was driven by the visual presentation of the objects, rather than the button pressing, we separately analyzed left and right AIP activation for trials in which the subject pressed a button (to indicate a “same” response in the size and pattern discrimination tasks) or withheld the button press (to indicate a “different” response). T-test contrasts on the % signal change extracted from ROIs showed no statistical difference between trials with button presses vs. those without for size and pattern discrimination in both left (size discrimination, p = 1; pattern discrimination, p = 1) and right AIP (size discrimination, p = 1; pattern discrimination, p = .4). Moreover, the signal change in both left and right AIP was significantly greater than zero, even for trials in which no button press occurred (p<.05 for all comparisons).

No areas were more activated for reaching than grasping, even at relatively liberal thresholds. This lack of reaching related activity is presumably due to the fact that both reaching and grasping included transport of the arm to the target location.

**Figure 7:**
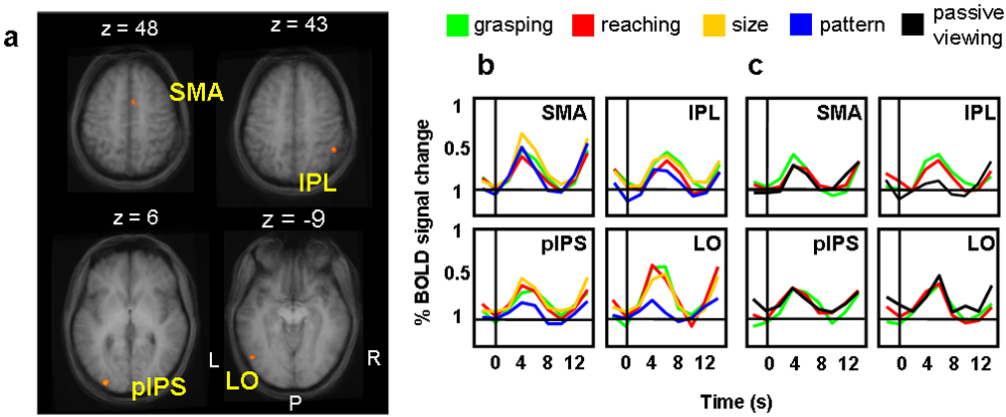
Group activation maps and percent BOLD signal change (% BSC) for size minus pattern discrimination in Experiment 1 and Experiment 2. Brain areas activated by comparing size versus pattern discrimination in Experiment 1: the left LO, the left posterior end of the IPS (pIPS), the right supplementary motor area (SMA) and the right inferior parietal lobe (IPL). The group activation map is based on the Talairach averaged group results shown on group-averaged for clarity on a single subject’s anatomical (which is not representative of sulcal patterns for all subjects). b) Event-related averaged time courses form each area for Experiment 1. c) Event-related averaged time courses for Experiment 2 in each of the brain regions localized by Experiment 1. Brain activation is measured in % BSC and time zero indicates visual stimuli onset. L = left, R = right, P = posterior.

**Figure 8:**
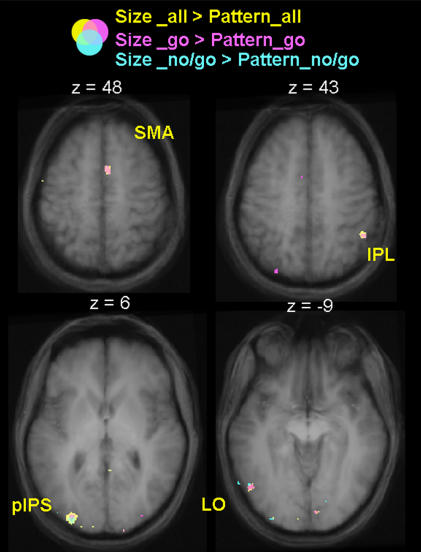
Overlaid activation maps for size versus pattern discrimination separated for go and no-go trials in Experiment 1. Brain areas activated for the comparison of size versus pattern discrimination in go trials (depicted in pink), no-go trials (depicted in light blue) and averaged go and no-go trials (depicted in yellow). When go trials and no-go trials were averaged, the contrast of size versus pattern discrimination produced activations in the left LO, the left posterior end of the IPS (pIPS), the right supplementary motor area (SMA) and the right inferior parietal lobe (IPL). When only go trials were taken into account, the contrast of size versus pattern discrimination produced quite similar activation in the left LO, the left posterior end of the IPS (pIPS), the right supplementary motor area (SMA) and the right inferior parietal lobe (IPL). When no/go trials alone were taken into consideration, the contrast of size versus pattern discrimination produced activations only in the right supplementary motor area (SMA) and the right inferior parietal lobe (IPL).

The voxelwise comparison of size discrimination vs. pattern discrimination revealed activation in left LO (Fig. 7a), as demonstrated by the ROI analyses in single subjects. In addition, this contrast revealed activation in the left posterior end of the IPS (pIPS), the right supplementary motor area (SMA) and the right inferior parietal lobe (IPL). All of these areas, including LO, showed a significant interaction for TASK and OBJECT FEATURES (see table 1 for statistical values) and post-hoc t-tests showed an equal response for grasping and reaching and a higher response for size vs. pattern discrimination in Experiment 1 (Fig. 7b). Most critically, no activation was observed in AIP. Although the activation in the IPL was near AIP, this area did not show greater activity for grasping than reaching, so it is unlikely to be involved in the visual preshaping of the hand. In addition, further analyses suggested that its greater activation in size vs. pattern discrimination was spurious. That is, in the right IPL, as well as the right SMA, the activation difference appeared to simply reflect the number of button presses made by the subjects using the left hand (contralateral to the right IPL). Specifically, the activation difference (in %BSC) between size and pattern discrimination was significantly correlated with the difference in the number of button presses between the two conditions for both the IPL (r = .672, p = .02) and the SMA (r = .75, p = .01). This was not the case in either LO (r = .142, p = .3) or the left pIPS (r = .071, p = .4). This interpretation was corroborated by further analyses of the fMRI data in which trials for size and pattern discrimination were divided into go and no/go responses (size discrimination/go, size discrimination/no-go, pattern discrimination/go, and pattern discrimination/no-go trials). Figure 8 shows overlaying activation maps elicited by comparing size versus pattern discrimination when go and no-go trials were averaged (size discrimination/all>pattern discrimination/all in yellow) and teased apart (size discrimination/go>pattern discrimination/go in pink, and size discrimination/no-go>pattern discrimination/no-go in light blue). We reasoned that if both go and no-go trials contributed to the activations found in LO, pIPS, IPL and SMA, then voxels from all three comparisons should appear in the vicinity of these areas. Conversely, if the activation in LO, pIPS, IPL and SMA was related to just the difference in the number of button presses, then only go trials should contribute to the overall cluster of activation. As predicted by the correlation analysis, we found that while only the go trials contributed to the activation of right IPL and right SMA, go trials and no-go trials contributed equally to the activations in left LO and left pIPS.

In addition, for Experiment 2, in all four areas (left LO, left pIPS, right SMA, right IPL) grasping, reaching, and passive viewing conditions did not differ from each other (Fig. 7c).

The opposite contrast, i.e. pattern vs. size discrimination did not produce any significant activation.

For both experiments, Talairach coordinates for the activated areas and p values for the relevant statistical comparisons are shown in Table 1.

## Discussion

Here we report evidence for a functional dissociation between areas within the dorsal and ventral streams when the size of graspable 3D objects was computed for guiding actions rather than for perceptual discrimination tasks. Area AIP in the dorsal stream, localized by grasping versus reaching, did not show any difference in activity between size and pattern discriminations, and conversely, area LO in the ventral stream, localized by size versus pattern discrimination, did not show any difference in activity for grasping versus reaching actions.

The role of AIP in the computation of object properties such as size during the selection of proper hand configuration is well-established in both the macaque [20], [52]–[54] and human brains [7]–[10], [19], [31], [55]–[58]. We now show that human AIP activation does not show any difference when size, a global object property, is computed for purely perceptual purposes, even with 3D objects. This suggests that AIP performs computations about object properties such as size only when those computations are relevant for planning hand actions. This conclusion is bolstered by other recent fMRI results showing that AIP processes both the shape of an object and the way it is grasped, both for action observation [17], [59] and for real grasping [60], and that AIP is not activated when object properties, including size, are necessary to pantomime an action beside the object’s location [44].

In Experiment 2, we also showed that human AIP responds to the presentation of grasapble 3D objects even when no action is planned. Given that macaque AIP is also active during the passive viewing of graspable objects, our data provide further support for proposals of functional equivalence between human AIP and macaque AIP [31], [61]. They also suggest that human AIP, like macaque AIP, contains neurons that are selective for shape, size, and orientation even during passive viewing [20]. The AIP response during passive viewing appeared comparable in magnitude to the response during the two perceptual tasks; though all three non-action tasks produced considerably smaller responses than the two action tasks. Perhaps AIP responds to the presentation of graspable 3D objects because they afford the potential to act, even when such actions are not explicitly required. We have recently found that another reach-related area in parietal cortex is modulated by the reachability of objects [62].

In contrast to AIP, left LO in the ventral stream appears to compute object properties when the task is purely perceptual, showing different levels of activation for size versus pattern discrimination only. On the other hand, during actions, left LO does not distinguish between conditions which require size computation (grasping) versus conditions which do not (reaching). Although it has been already established that activation in LO is higher for objects than patterns [6] and equal for grasping and reaching [9], the use of real 3D graspable objects in our study is novel. The activation for size vs. pattern discrimination was not observed throughout the entire LOC, but rather was limited to the most superior and lateral division (LO) and appeared here only in the left hemisphere. Size-selective activation is consistent with the recent proposal that LO is more concerned with global form (e.g., shape, orientation and size) than material properties [e.g., color and texture, 12,28]. In addition, we found that LO activation was equivalent for passive viewing, grasping, and reaching suggesting that the response in all three conditions is simply due to the visual presentation of a coherent object. This visual response does not appear to be necessary for accurate grasping, given that two patients can still perform accurate grasping, even in the complete absence of LO [DF: 12] or almost the entire occipito-temporal visual pathway bilaterally [SB: 63].

Several features of the activation pattern in LO struck us as surprising. First, although we would have predicted that the response to size discrimination would have been higher than pattern discrimination, grasping and reaching, we found instead that size discrimination, grasping and reaching were all higher than pattern discrimination. A likely explanation is that size discrimination, grasping and reaching all involve attention to the global object properties; whereas, the pattern discrimination requires attention to the local intrinsic detail. Second, we were somewhat surprised that significant activation for size (vs. pattern) discrimination was observed in only left LO but not right LO. Two prior studies suggest that the distinction between global and local object processing is bilateral [28], [29] and there is a well-known distinction between the left and right hemispheres in local and global processing, respectively [64], [65]. It is possible that the distinction is due to the use of real 3D stimuli, though formal testing would require a direct comparison between 2D and 3D stimuli. Third, based on the proposed subdivisions of LO, we were also surprised not to find significantly greater activation in VOT for pattern vs. size discrimination. This may be related to the fact that our patterns did not convey a sense of texture or the material properties of the objects.

In addition to AIP, results from our voxelwise contrast of grasping vs. reaching revealed a network of areas, mostly in left parietal cortex. These regions included S1, sPCS, hIPS, and PO, all in the left hemisphere, as well as early visual areas in medial occipital cortex bilaterally. The activity in S1 is likely related to the additional tactile stimulation of the fingers during grasping. Indeed, while AIP responded to passive visual stimulation (as expected from macaque neurophysiology), S1 did not. Prior work has discussed the difficulty of distinguishing AIP from nearby somatosensory regions, especially S1 [9], [31]. Here this distinction was facilitated by the introduction of a purely visual condition (passive viewing) which activates AIP but not S1.

The activation of a network of parietal areas more responsive to grasping than reaching shows that AIP does not work alone. The specific and causal role of AIP in hand pre-shaping for grasping (and thus its sensitivity to object size) is supported by recent TMS literature [56], [57], [66] showing that TMS over area AIP, but not over PO or caudal IPS, selectively modulated on-line handgrip. These additional areas might, however, be involved in ancillary visuomotor processes. For example, the activation in the sPCS may correspond to parietal area 5 [47], which could be involved in computing the spatial position of the hand [67], [68] and/or using proprioception to guide action [69]–[71]. Activation in the vicinity of hIPS has been reported in association with 3D feature processing [51], [55], [72]–[74]. This region (area cIPS) may be a homologue of macaque cIPS [51], [55], [72] which codes object features and send projections to AIP [54]. Another possibility is that hIPS corresponds to human LIP [75], which may relay inputs from macaque V3A to AIP [76]. Activation in the left PO has been previously reported for grasping [77] and pointing [78]–[80]. This region in humans may be homologous with a monkey area in the anterior bank of PO (V6A), in which a sub-population of neurons selectively responds when the monkey attends to, reaches towards, or grasps an object [81]. In sum, although our analyses of grasping vs. reaching focused on AIP, there are several parietal areas in the dorsal stream that show a similar response pattern and likely form a network involved in the sensorimotor control of grasping.

Besides left LO, object size discrimination activated the inferior pIPS in the left hemisphere and the IPL and SMA in the right hemisphere. We suggested that the activations found in the right hemisphere may arise from a greater demand in term of arbitrary stimulus-response association and motor preparation processes, given the greater number of button presses made by the contralateral left hand in the size discrimination task [82]. This response-related hypothesis is also supported by the lack of significant activation in those two areas for the passive viewing condition in Experiment 2 (Table 1). Conversely, the pIPS activation was ipsilateral to the hand used for discrimination button presses, was not related to the number of button presses, and showed a significant response to objects during passive viewing. Object-selective activation in the vicinity of pIPS is well-established [83], [84] though less widely described than activation in LO.

### Conclusions

The strength and the novelty of our findings comes chiefly from i) the clear functional dissociation we found in the same subjects, in the same experiment and using the same 3D objects, ii) the visual control condition enabling us to better define the visual properties of human AIP and to distinguish the location of AIP from the adjacent somatosensory cortex with higher accuracy. Although visually-guided grasping of 3D objects requires processing of object size, that computation appears to rely on different neural mechanisms than those involved in the perceptual discrimination of size. AIP showed a response during both size and pattern discrimination, consistent with its activation during passive viewing, but there was no differential response between these two conditions. Conversely, left LO showed a response during both grasping and reaching, but no difference in the magnitude of this response, consistent with its activation to the visual presentation of any object. Taken together these results support a dual representation of objects for the purposes of action and perception in neurologically intact human subjects. In other words, the human visual system does not construct a single representation of the world for both visual perception and the visual control of action. Instead, areas in the ventral stream mediate the visual perception of objects whereas areas in the dorsal stream mediate the visual control of action directed at those same objects.
